# Carbon Dots for Nucleic Acid-Based Diagnostics and Therapeutics: Focus on Oxidative DNA Damage

**DOI:** 10.3390/ijms26168077

**Published:** 2025-08-21

**Authors:** Barbara Pascucci, Maria Moccia, Mariarosaria D’Errico, Fabrizio Vetica, Michele Saviano, Francesca Leonelli, Annalisa Masi

**Affiliations:** 1 Institute of Crystallography, Department of Chemical Sciences and Materials Technologies, National Research Council of Italy, Strada Provinciale 35d, 9, Montelibretti, 00010 Rome, Italy; barbara.pascucci@cnr.it (B.P.); maria.moccia@cnr.it (M.M.); 2 Department of Environment and Health, Istituto Superiore di Sanità, Viale Regina Elena 299, 00161 Rome, Italy; mariarosaria.derrico@iss.it; 3 Dipartimento di Chimica, Università degli Studi di Roma “La Sapienza”, Piazzale Aldo Moro 5, 00185 Rome, Italy; fabrizio.vetica@uniroma1.it; 4 Institute of Crystallography, National Research Council of Italy, URT Caserta, via Vivaldi 43, 81100 Caserta, Italy; michele.saviano@cnr.it

**Keywords:** nucleic acid, carbon dots, oxidative DNA damage, disease diagnostics

## Abstract

Carbon dots (CDs) are gaining significant attention as multifunctional nanomaterials due to their optical properties, aqueous dispersibility, redox activity, and overall biocompatibility. This review presents a critical overview of the recent advances concerning the application of CDs in nucleic acid-centered diagnostics, with a specific focus on oxidative DNA damage. The use of CDs for the detection of oxidative DNA damage biomarkers, such as 8-oxo-2′-deoxyguanosine (8-oxo-dG), and their potential roles as fluorescent probes in environments related to oxidative stress is discussed in detail. The relationship between surface functionalization and biological performance is examined, highlighting how physicochemical properties dictate both the beneficial and adverse biological responses to CDs. Remarkably, CDs can act as antioxidants, mitigating oxidative damage, or as pro-oxidants, inducing cytotoxic effects, an ambivalent behavior that can be strategically harnessed for cytoprotection or selective tumor cell killing. Overall, this review outlines how CDs can contribute to the development of precision tools for studying oxidative environments affecting nucleic acids, with important implications for both diagnostics and redox-based therapeutic strategies of human diseases.

## 1. Introduction

Oxidative DNA damage arises from the interaction between nucleic acids and reactive oxygen species (ROS), which are generated through mitochondrial respiration, inflammatory processes, and environmental stressors such as ionizing radiation, pollutants, and xenobiotics [1,2,3].

Oxidative DNA damage occurs in many forms, including modifications to the DNA bases, strand breaks, and DNA–protein crosslinks. If not properly repaired, these types of damage can lead to base-pairing errors, genomic instability, and mutations, factors that are closely linked to the development of serious health conditions like cancer, neurodegenerative diseases, and disorders related to aging. Due to their biological relevance, the detection and quantification of oxidative DNA damage has become essential for understanding disease mechanisms and developing diagnostic strategies for diseases.

To address this need for innovative diagnostic strategies for diseases, researchers have been exploring novel sensing and therapeutic platforms, among which carbon dots (CDs) have gained particular attention due to their unique combination of photoluminescence, redox activity, biocompatibility, and functional versatility [4,5,6,7,8,9,10,11,12,13]. Because CDs can interact with DNA, researchers have developed innovative tools to detect oxidative DNA damage and assess the redox state of specific cellular regions. Moreover, depending on their physicochemical properties, CDs can exhibit both antioxidant and pro-oxidant behavior, an ambivalent feature that can be strategically harnessed for cytoprotection or selective tumor cell killing [14,15].

This review explores the use of CDs in the context of nucleic acid-based diagnostics and therapeutics, with a particular emphasis on oxidative DNA damage. We begin by outlining the definition, synthesis methods, and characterization techniques of CDs, offering the necessary background to understand their functions and interactions within biological systems. We then discuss the mechanisms and biological consequences of oxidative DNA damage, followed by an in-depth analysis of the redox duality of CDs. Finally, we highlight their applications in the detection and therapeutic modulation of oxidative damage to nucleic acids.

## 2. Carbon Dots: Definition, Synthesis, and Characterization

### 2.1. Classification and Structural Features

**Figure 1:**
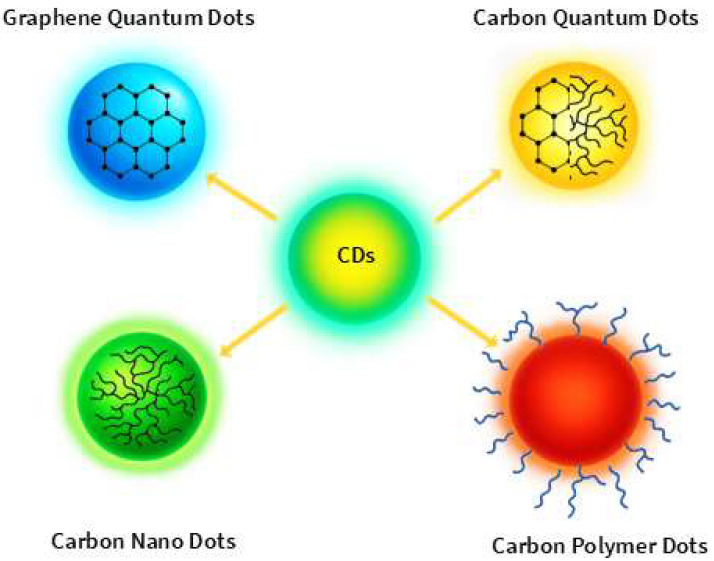
Schematic representation of the main categories of CDs: Graphene Quantum Dots, Carbon Quantum Dots, Carbon NanoDots, and Carbon Polymer Dots.

CDs are a highly versatile class of nanomaterials, despite their structural diversity. The term “carbon dots” refers to a broad range of fluorescent, quasi-spherical nanoparticles typically smaller than 10 nm, which differ significantly in terms of crystallinity, composition, and synthetic methods. Based on these distinctions, CDs are generally categorized into four main subtypes: Graphene Quantum Dots (GQDs), Carbon Quantum Dots (CQDs), Carbon NanoDots (CNDs), and Carbon Polymer Dots (CPDs) (Figure 1). GQDs are nanometer-thin carbon materials whose optical properties are strongly influenced by the confinement of electrons in a small space (quantum confinement) and by localized energy states introduced at the edges of the graphene lattice (edge-state effects), both of which contribute to their fluorescence [16]. CQDs are quasi-spherical and feature a graphitic or semi-crystalline core [17,18]. CNDs, in contrast, are amorphous and typically exhibit fluorescence arising from surface or molecular fluorophores [19]. CPDs combine carbonaceous domains with partially retained polymeric chains and often derive their emission from crosslinked structures or residual organic functionalities [20].

A central aspect of CD architecture lies in their surface functionalization. CDs possess oxygenated and nitrogenated groups such as –OH, –COOH, and –NH_2_, which are crucial for aqueous stability, bioconjugation, and interaction with biological molecules. These groups also influence fluorescence, redox behavior, and the ability to generate or scavenge ROS, making CDs highly responsive to their chemical environment. Specific oxygen functionalities (e.g., carbonyls, phenols, carboxyls) have been implicated in the redox mechanisms of CDs and their ROS modulation capacity [14].

Further control over CD properties is achieved via heteroatom doping, which involves the incorporation of atoms such as nitrogen, sulfur, phosphorus, or halogens into the carbon framework. Nitrogen doping, for example, enhances quantum yield and electron-donating capacity through the formation of defect-related emissive states. Chlorine and phosphorus doping have also been shown to significantly increase ROS generation and fluorescence lifetime, enabling potential use in oxidative stress sensing and photodynamic therapy [14].

### 2.2. Synthesis Strategies and Characterization Techniques

**Figure 2:**
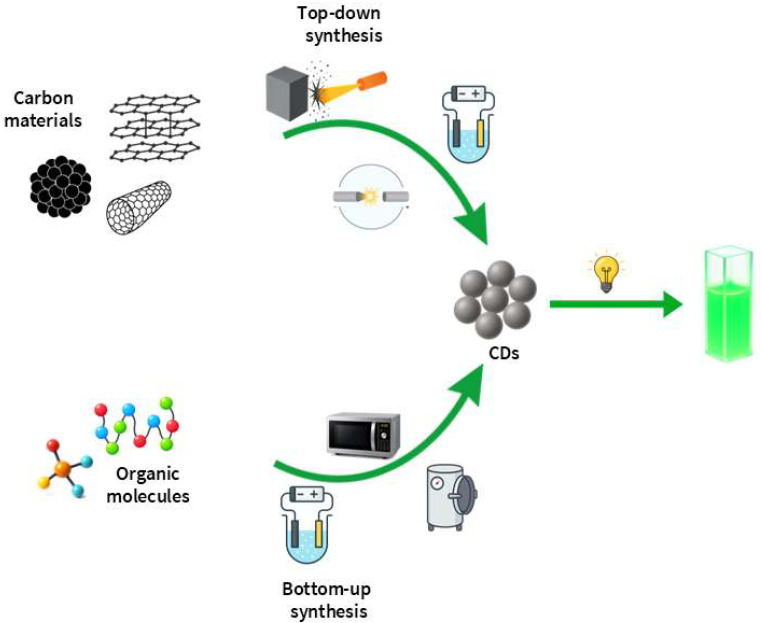
Schematic representation of top-down and bottom-up synthesis routes for CDs. Top-down methods break down carbon materials, while bottom-up approaches carbonize organic precursors, both leading to fluorescent CDs.

The synthetic approach used plays a pivotal role in determining both the morphology and functionality of CDs. Top-down methods are based on the fragmentation of bulk carbon sources (e.g., graphite, carbon nanotubes, carbon black), often via harsh chemical or electrochemical treatments (e.g., laser ablation, arc discharge). These methods are primarily used for GQDs but tend to produce lower yields and more structural defects. On the other hand, bottom-up methods, including hydrothermal carbonization, pyrolysis, and microwave-assisted synthesis, are favored for their simplicity, tunability, and compatibility with a wide variety of organic precursors. These approaches enable control over particle size, surface groups, and optical properties, and are generally more scalable and sustainable [18,21] (Figure 2).

The physicochemical properties of CDs are investigated using a combination of advanced analytical techniques. Morphological and dimensional analysis is typically performed using transmission electron microscopy (TEM) and atomic force microscopy (AFM), which provide nanoscale resolution of particle size and shape.

Spectroscopic techniques, such as Fourier-transform infrared spectroscopy (FTIR), X-ray photoelectron spectroscopy (XPS), and Raman spectroscopy, are employed to identify surface functional groups and elemental composition. Optical behavior is studied through UV-Vis absorption, steady-state and time-resolved photoluminescence, and quantum yield measurements. Advanced techniques such as single-particle fluorescence spectroscopy [22,23] and transient absorption (TA) spectroscopy have been essential for understanding how CDs emit light and for identifying trap states. Trap states are small imperfections or special sites inside the material that can temporarily hold electrons and affect how and when light is released and are present especially in doped CDs [24,25].

Colloidal characterization methods such as dynamic light scattering (DLS) and zeta potential analysis are essential to evaluate size distribution and surface charge under physiological conditions. These parameters are crucial for ensuring colloidal stability and biological compatibility, especially in imaging and therapeutic applications.

## 3. CDs and Oxidative DNA Damage

### 3.1. Mechanism and Biological Relevance of Oxidative DNA Damage

**Scheme 1:**
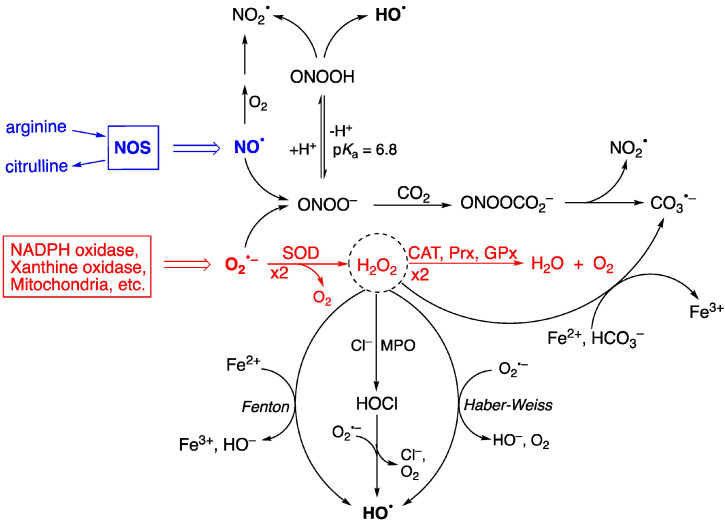
Schematic overview of the key chemical pathways involved in the generation of the reactive oxygen species (ROS). Copyright 2025, The Authors, published by MDPI [26].

Oxidative DNA damage occurs when ROS interact with DNA, altering its chemical structure and integrity. ROS are chemically reactive molecules derived from oxygen, which include free radicals like hydroxyl radicals (HO•), superoxide anion (O_2_•^−^), and non-radical oxidants such as hydrogen peroxide (H_2_O_2_) (Scheme 1) [26].

**Figure 3:**
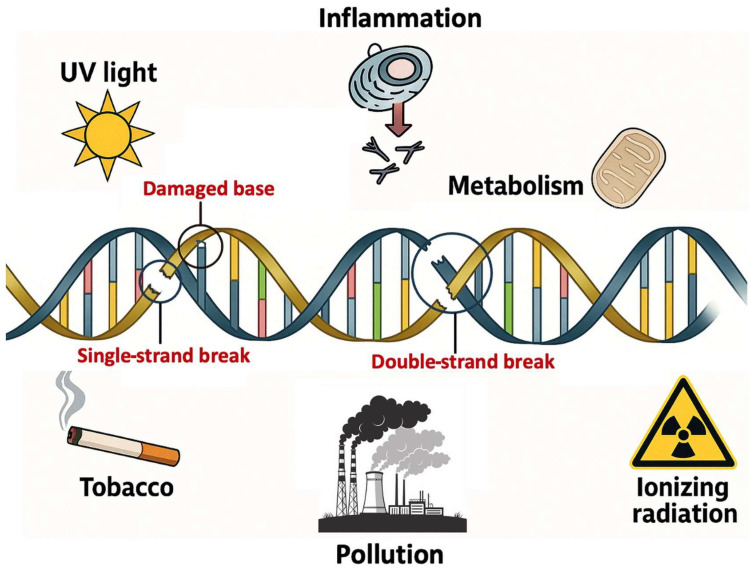
Endogenous and exogenous sources of oxidative stress leading to DNA damage.

These reactive species are byproducts of normal cellular metabolism and are also generated in response to external environmental insults (Figure 3). The balance between ROS production and antioxidant defenses determines the extent of oxidative stress and its impact on cellular components, including DNA [27]. Endogenously, the mitochondrial electron transport chain represents the primary source of ROS during cellular respiration. In this process, the incomplete reduction of oxygen leads to the formation of superoxide radicals. Inflammatory responses also contribute significantly to ROS production, as activated immune cells like neutrophils and macrophages release superoxide and hydrogen peroxide to combat pathogens. Enzymatic reactions catalyzed by xanthine oxidase, cytochrome P450 enzymes, and others further increase intracellular ROS levels [28].

Exogenously, ionizing radiation, ultraviolet (UV) light, and exposure to environmental pollutants like cigarette smoke, heavy metals, and industrial chemicals can significantly increase ROS levels. These factors either directly generate ROS or catalyze their production through chemical reactions involving molecular oxygen [29].

ROS are highly reactive and interact with DNA, causing modifications to nitrogenous bases, the sugar–phosphate backbone, and even creating crosslinks between DNA strands or with proteins. Among these interactions, base oxidation is the most extensively studied due to its mutagenic potential. Guanine, being the most easily oxidized base, is particularly vulnerable. One of the hallmark products of guanine oxidation is 8-oxoguanine (8-oxoG), which can mispair with adenine during replication, leading to G:C to T:A transversions, a type of mutation commonly associated with cancer [30,31]. Similarly, cytosine can be oxidized to form 5-hydroxycytosine, while adenine and thymine can undergo oxidation to produce 8-hydroxyadenine and thymine glycol, respectively [32].

HO• radicals, generated via Fenton and Haber–Weiss reactions (Scheme 1), represent one of the most reactive ROS and attack both DNA bases (85–90%) and the sugar moiety (10–15%). Their action on the C5′ position of 2′-deoxyribose leads to the formation of tandem lesions known as 5′,8-cyclo-2′-deoxyadenosine (cdA) and 5′,8-cyclo-2′-deoxyguanosine (cdG), known as cyclopurines (cPu). These adducts result from an intramolecular bond between C5′ of the sugar and C8 of the purine base, exist as 5′R and 5′S diastereomers, and are highly stable, helix-distorting lesions that obstruct both DNA and RNA polymerase. In addition to cPu formation, hydrogen abstraction from the sugar moiety destabilizes the phosphodiester backbone, resulting in single- or double-strand breaks. Strand breaks contribute to genomic instability, increasing the likelihood of chromosomal rearrangements or loss of genetic material. If these lesions are not repaired promptly, they can activate cell death pathways, leading to apoptosis or cellular senescence. In addition to these effects, oxidative stress can create abasic (AP) sites by removing bases from the sugar-phosphate backbone, which disrupts replication and transcription processes [29].

On a broader scale, oxidative DNA damage plays a central role in aging and the pathogenesis of various diseases, including neurodegenerative disorders and cardiovascular conditions, in addition to cancer [33,34].

To counteract the harmful effects of ROS, cells have evolved intricate DNA repair mechanisms. The base excision repair (BER) pathway is the primary mechanism for repairing oxidative lesions. This process is initiated by DNA glycosylases, which recognize and excise damaged bases. For example, the enzyme OGG1 specifically removes 8-oxoG, creating an abasic site that is subsequently processed by an AP endonuclease. The resulting gap is filled and ligated by DNA polymerase β and DNA ligase I, restoring the DNA sequence [35].

Other repair pathways also play roles in addressing oxidative DNA damage. Nucleotide excision repair (NER) can remove bulky oxidative lesions [36], while mismatch repair (MMR) ensures the fidelity of replication by correcting mismatched base pairs caused by oxidative lesions In some cases, direct reversal mechanisms, such as the activity of alkylguanine DNA alkyltransferase (AGT), can directly repair specific oxidative lesions without excision [37,38,39].

The study of oxidative DNA damage has profound implications for understanding disease mechanisms and developing therapeutic strategies. Elevated oxidative stress is a hallmark of cancer, where mutations induced by oxidative damage can activate oncogenes or inactivate tumor suppressor genes. Similarly, in neurodegenerative diseases like Alzheimer’s and Parkinson’s, oxidative DNA damage in neurons contributes to cell death and functional decline [40]. Strategies aimed at enhancing antioxidant defenses or modulating DNA repair pathways are being explored as potential therapeutic approaches.

### 3.2. Redox Duality of Carbon Dots

#### 3.2.1. Pro-Oxidant and Cytotoxic Effects of Carbon Dots

**Figure 4:**
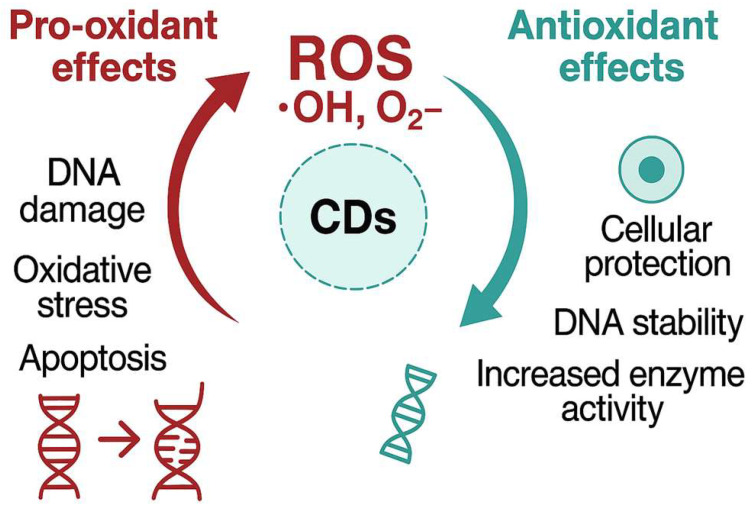
Schematic representation of the dual roles of CDs showing antioxidant (green) and pro-oxidant (red) effects, including ROS scavenging, cellular protection, DNA damage, and apoptosis.

CDs represent a new generation of carbon nanomaterials which show great promise for drug delivery, imaging, and nanomedicine applications [41,42]. However, their interactions with biological macromolecules, particularly DNA, remain a crucial area of investigation due to their dual antioxidant and pro-oxidant behaviors that creates both valuable and potentially hazardous properties (Figure 4) [14].

Their structural and chemical composition, including the presence of heteroatoms such as nitrogen, sulfur, or oxygen, influence their optical and electronic properties and, consequently, their ability to generate or neutralize ROS [43,44].

The cellular reaction to CDs is greatly influenced by surface chemistry [45]. The safest modification of CDs exists in PEG-modified CDs (CDs-PEG) since these particles show no harmful effects on cellular structure or function at any concentration level. The pristine CDs (CDs-Pri), namely carbon dots without surface functionalization, demonstrated non-toxic properties but caused oxidative stress and cell cycle disruption at certain concentrations which led to increased cell proliferation. The most toxic were polyethyleneimine-coated CDs (CDs-PEI) as they were able to penetrate the nucleus and disrupt cell division, leading to high cytotoxicity (Table 1) [46].

Cell-type affects the impact of CDs, with some cell lines tolerating their presence while others exhibit significant cytotoxicity [45].

Additionally, light exposure plays a crucial role in CD toxicity. It has been demonstrated that photodegradation of CDs can result in the formation of smaller cytotoxic molecules that adversely affect human cells. These findings emphasize the importance of taking into account environmental factors, like light exposure, when evaluating CD safety [53]. One of the primary mechanisms of CD-induced toxicity is the generation of ROS, leading to oxidative stress, mitochondrial dysfunction, lipids and protein damage, and DNA strand breaks, ultimately resulting in apoptosis or necrosis [54], or through the inhibition of key antioxidant enzymes. In particular, certain CDs have been shown to acidify their surrounding microenvironment, thereby suppressing peroxidase activity, increasing oxidative stress and compromising DNA repair mechanisms [15]. This effect has significant implications for both antibacterial resistance and cancer growth.

Interactions of CDs with the environment must also be carefully evaluated. For example, iron-doped CDs (CDs–Fe) strongly bind to arsenate and increase its bioaccumulation in earthworms, while simultaneously inducing mitochondrial DNA damage and oxidative stress. Arsenate further intensifies this damage by inhibiting catalase (CAT), highlighting the potential ecological risks of CDs and their ability to reduce arsenic cycling [55]. At a molecular level, CDs have been shown to impair BER, particularly by interfering with the ligation step, which is crucial for repairing oxidative DNA lesions. This disruption may lead to the accumulation of mutations and increased genomic instability [15]. Recent evidence also highlights the role of chirality in mediating CD–cell interactions, with chiral carbon dots showing enantioselective accumulation in subcellular compartments and differential affinity for nucleic acids, suggesting that stereochemical configuration may influence both targeting and toxicity profiles [56,57].

#### 3.2.2. ROS Scavenging and Protective Roles of CDs

Although some CDs have demonstrated cytotoxic and genotoxic potential, especially under photoactivation or at high concentrations, a growing subset of studies highlights their protective role against oxidative stress (Figure 4). This duality is closely linked to their structural and chemical features, including doping elements, surface passivation, and the nature of functional groups.

**Figure 5:**
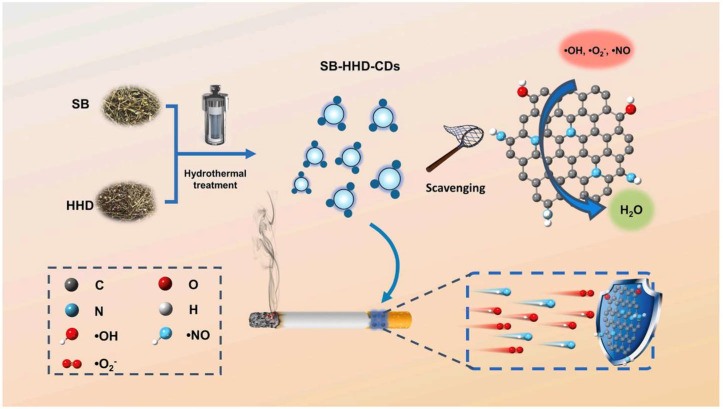
Schematic representation of the synthesis of SB-HHD-derived carbon dots (SB-HHD-CDs) via hydrothermal treatment and their subsequent application in cigarette filters. The figure illustrates the ROS-scavenging capacity of the CDs, including neutralization of •OH, •O_2_^−^, and H_2_O_2_ species. Copyright (2023), reprinted from [59] with permission from Elsevier. License number 6067240448060.

Antioxidant CDs can effectively scavenge reactive oxygen and nitrogen species, offering protection against DNA oxidation and cellular damage [58]. Their flexible redox behavior has made them attractive candidates for therapeutic strategies aimed at restoring redox balance, particularly in diseases associated with oxidative stress. Mechanistically, the ROS-scavenging ability of CDs is attributed to several molecular pathways. First, CDs with electron-rich functional groups (e.g., hydroxyl, amine, or carboxyl) can act as direct electron donors, reducing ROS such as O_2_•^−^, hydroxyl radical HO•, or singlet oxygen (^1^O_2_) into less reactive species. In addition, doped heteroatoms such as nitrogen or sulfur enhance the electron delocalization across the CD surface, increasing their redox buffering capacity. These effects are especially pronounced in CDs synthesized from biomolecules rich in thiol or aromatic groups, which stabilize radical intermediates during ROS quenching. Gao and co-workers developed promising CDs synthesized from *Scutellaria barbata* and *Herba Hedyotis diffusae* (SB-HHD-CDs). These CDs showed remarkable stability under varied pH and temperature conditions and effectively neutralized multiple reactive oxygen and nitrogen species (RONS) even at low concentrations. When incorporated into cigarette filters, they removed over 80% of RONS produced during tobacco combustion, underscoring their potential in both biomedical and environmental applications (Figure 5) [59].

Additional studies suggest that CDs may exert indirect antioxidant effects by modulating endogenous defense systems. For instance, Innocenzi et al. demonstrated that CDs derived from natural antioxidants, such as polyphenols and plant extracts, enriched in redox-active functional groups (e.g., hydroxyl and carboxyl moieties), were able to support the activity of cellular antioxidant enzymes like superoxide dismutase (SOD), catalase, and glutathione peroxidase (GPx). These properties are particularly relevant in chronic inflammatory conditions and skin barrier repair, where redox imbalance plays a central role [14].

These findings suggest that the antioxidant action of CDs involve complex interactions with intracellular signaling pathways and redox-sensitive transcription factors such as Nrf2. Indeed, Zhou et al. found that CDs encapsulated within metal–organic frameworks (CDs@ZIF-8) enhance Nrf2 nuclear translocation and increase expression of downstream antioxidant enzymes like HO-1 and NQO1, consistent with activation of the Nrf2/ARE pathway [60]. CDs derived from natural or biocompatible sources, such as ascorbic acid, citric acid, or amino acids, have shown potent antioxidant activity both in vitro and in vivo. For instance, Wang et al. reported CDs synthesized from glutathione and polyethylene glycol exhibiting concentration-dependent scavenging activity against DPPH and ABTS radicals, along with cytoprotection in oxidative stress-challenged cells [61].

The same study also reported that CDs derived from folic acid precursors exhibited strong antioxidant activity while maintaining low cytotoxicity in fibroblasts and keratinocytes, supporting their use as protective agents in dermatological formulations exposed to oxidative insults [61].

## 4. Applications of Carbon Dots in Oxidative DNA Damage Detection and Therapy

CDs are increasingly recognized for their ability to interface with nucleic acids through multiple non-covalent interactions, including electrostatic attraction, groove binding, and intercalation, or even covalent conjugation depending on their surface charge, size, and functional groups [57]. These characteristics have long supported their use in biomedical applications such as biosensing, nucleic acid delivery, and fluorescence imaging [62,63]. In recent years, interest in the use of CDs in the context of oxidative DNA damage has grown, although it remains a relatively underexplored area of research. Thanks to their unique properties, CDs are being investigated as versatile tools, from optical probes for oxidatively damaged DNA [64], to scavengers of reactive oxygen species in antioxidant therapies [59], and even as pro-oxidant agents capable of inducing selective genotoxic stress in tumor cells through photodynamic and chemodynamic mechanisms [65,66,67,68]. These multifunctional properties place CDs as promising platforms for developing nanotechnologies that integrate diagnostic and therapeutic capabilities in the context of redox imbalance and DNA integrity.

### 4.1. Detection of Oxidative DNA Damage Biomarkers

Early detection of oxidative DNA damage is critical for diagnosing and monitoring diseases characterized by redox imbalance, including cancer, neurodegenerative disorders, and chronic inflammation [69]. A promising strategy involves the indirect sensing of oxidative stress by detecting ROS, which are known to precede and promote nucleic acid oxidation. For instance, boronic acid-functionalized CDs undergo selective fluorescence quenching in the presence of H_2_O_2_, enabling their use as intracellular probes to visualize oxidative microenvironments. In RAW264.7 macrophages stimulated with lipopolysaccharide, these probes revealed nuclear accumulation of H_2_O_2_, reflecting early oxidative signaling before overt DNA damage [70]. These measurements are particularly relevant given the established contribution of ROS to the formation of oxidative DNA lesions, including 8-oxo-dG, 8-hydroxy-2′-deoxyguanosine (8-OHdG), and cPu [69,71], markers of oxidative DNA damage in both nuclear and mitochondrial genomes. Clinical studies have consistently reported elevated urinary or serum levels of these biomarkers in association with disease onset and progression, notably in colorectal, breast, and lung cancer, as well as in cardiovascular and neurodegenerative conditions [72,73,74].

**Figure 6:**
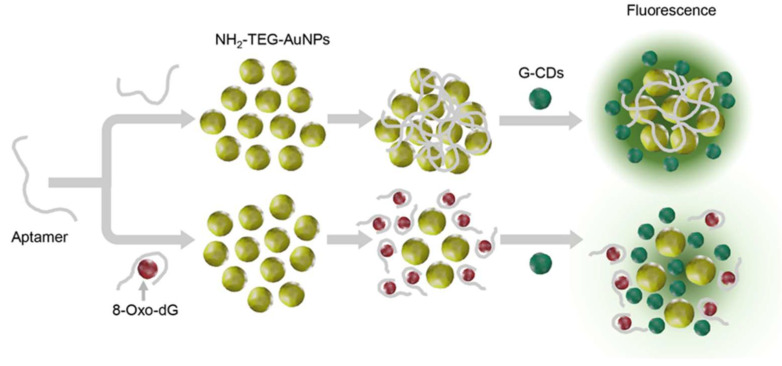
Schematic representation of an aptamer-based CD sensor for 8-oxo-dG detection, exploiting fluorescence quenching of G-CDs by AuNPs and signal recovery upon target binding. Copyright (2023), with permission from American Chemical Society [75].

Drawing on their unique optical properties, including tunable fluorescence and photoluminescence, and favorable biocompatibility, CDs have been integrated into biosensor platforms for the highly specific detection of these lesions. These applications support the potential of CD-based technologies in the development of diagnostic tools for redox-related pathologies. In an aptamer-based system reported by Ngernpimai et al., green-emitting CDs were combined with amine-functionalized gold nanoparticles (AuNPs) and a DNA aptamer selective for 8-oxo-dG. The interaction between the aptamer and target induces nanoparticle aggregation, modulating the fluorescence signal through an inner-filter effect (Figure 6). The assay achieved a detection limit of 15.89 nM and was validated in synthetic urine samples [75].

An alternative detection modality was developed by Ye et al., employing a FRET configuration based on CDs immobilized on nanoporous alumina membranes and silver nanoparticles encapsulated in zeolitic imidazolate framework-8 (Au@ZIF-8) as quenchers. The approach allowed highly sensitive detection of 8-OHdG, with a limit of 0.31 nM, and demonstrated robust performance in real urine matrices [76].

Electrochemical sensors incorporating CDs have also shown excellent performance in quantifying oxidative lesions. Zhang et al. described a nanohybrid comprising carbon dots encapsulated in platinum nanocrystals on nitrogen-doped graphene, which exhibited electrocatalytic properties for the detection of 8-OHdG in DNA hydrolysates and urine, with a detection threshold below 1 nM [77]. Additionally, Kudra et al. used CDs in a fluorescence resonance energy transfer (FRET)-based system to monitor DNA strand alterations induced by UV and HO•. Changes in fluorescence were correlated with structural perturbations in λ-DNA, suggesting that CD–DNA interactions can provide indirect but sensitive readouts of DNA damage accumulation [64].

### 4.2. Carbon Dots in Cancer Therapy: Pro-Oxidant Strategies for Selective Tumor Targeting

The capacity of CDs to modulate redox processes has led to their emerging use in cancer therapy, where their pro-oxidant behavior can be exploited to selectively induce cytotoxic stress in tumor cells. Owing to the intrinsic redox imbalance of many cancer cells, which often display elevated basal ROS levels, further induction of oxidative stress by CDs can tip the balance toward apoptosis or necrosis. As previously discussed in Section 3.2.1, certain CDs can promote intracellular oxidative stress through ROS generation and redox cycling. Building on this pro-oxidant activity, recent studies have explored their therapeutic exploitation in cancer, where selective oxidative injury to tumor cells can be achieved via photodynamic, chemodynamic, or catalytically driven redox strategies [59,78,79].

In photodynamic therapy (PDT), CDs act as photosensitizers that generate ^1^O_2_ or HO• upon light irradiation, leading to localized oxidative damage. Their small size, tunable emission, and biocompatibility make them ideal for deep-tissue imaging and simultaneous therapy. In particular, compared to more traditional nanomaterials such as TiO_2_ nanoparticles or porphyrin-based photosensitizers, CDs offer enhanced photostability and excitation in the visible to near-infrared range, enabling effective ROS production with lower off-target phototoxicity. These characteristics, coupled with their intrinsic fluorescence, render CDs particularly attractive for theranostic applications. For instance, ultrasmall CDs have been shown to induce DNA damage and cell cycle arrest in breast cancer cells, while cationic CDs selectively accumulate in cancer cell nuclei, triggering apoptosis via oxidative DNA lesions [65,66,67].

In contrast, chemodynamic therapy (CDT), exploits the catalytic properties of CDs, especially those doped with redox-active metals, to promote Fenton-like reactions in the tumor microenvironment. While iron oxide nanoparticles [80] are widely studied for CDT, their tendency to aggregate and limited catalytic efficiency in mildly acidic tumor pH can restrict efficacy. CDs overcome some of these limitations through their tunable surface chemistry and enhanced dispersibility, which support more efficient ROS generation. In a representative example, ginseng-derived CDs significantly enhanced ferroptosis cell death in squamous carcinoma by increasing intracellular ROS, downregulating phospholipid glutathione peroxidase (GPX-4), and upregulating cyclooxygenase-2 (COX-2), effects often not achieved by conventional iron-based systems [81].

Further, co-delivery systems incorporating CDs and chemotherapeutic agents, such as doxorubicin or cisplatin, have demonstrated synergistic oxidative damage through both DNA intercalation and CD-induced ROS amplification. This approach has parallels with polymeric nanoparticles [68] and Metal–Organic Frameworks [82] used for redox-modulated drug delivery, but CDs offer unique advantages in terms of smaller size, enhanced tumor penetration, and real-time optical monitoring. Fluorescent CDs functionalized with cisplatin (IV), doxorubicin, and targeting ligands (e.g., folic acid, RGD) exhibited enhanced selectivity and cytotoxicity in tumor cells compared to free drugs or standard nanocarriers [83,84,85].

In recent work of Priyadarshini and co-workers, CD-based organometallic nanoconjugates such as silver@CDs (Ag@CDs) demonstrated potent cytotoxic effects against HeLa cancer cells, largely mediated by ROS production, as shown through fluorescence imaging. In contrast, gold@CDs (Au@CDs) and unmodified CDs exhibited much lower toxicity, indicating Ag@CDs’ potential as anticancer agents [65]. Further, as shown by Şimşek et al., CDs derived from Nerium oleander extracts selectively induced DNA damage in MCF-7 breast cancer cells, with minimal impact on normal dermal fibroblasts. The comet and micronucleus assays confirmed significant genomic instability, likely driven by oxidative stress and the high-oxygenated functional group content on the CDs’ surface [66]. Similar effects were observed in studies on intracellular trafficking, where cationic CDs caused DNA damage, altered cell cycles, and accumulated in the nuclei of cancer cell lines [67].

## 5. Challenges and Future Directions

Despite the remarkable progress in understanding and exploiting the redox properties of CDs, several key challenges must be addressed to enable their clinical application in nucleic acid-based diagnostics and redox therapies. A principal limitation remains the intrinsic heterogeneity of CDs arising from variations in synthetic methods, precursors, and post-synthetic modifications. This variability affects their physicochemical properties, including size, charge, quantum yield, and surface functionality, complicating the reproducibility of biological studies and the design of standardized therapeutic protocols [14].

Another key challenge concerns the dual redox activity of CDs, depending on their structure, doping elements, and cellular environment. While their capacity to scavenge or generate ROS is central to their biomedical utility, it also poses a toxicity risk in non-target tissues, particularly when redox balance is not closely controlled [58]. In this context, the rational design of CDs with environment-sensitive or externally activatable redox profiles (e.g., via pH, enzymatic activity, or light) is an attractive but underexplored strategy. The long-term biodistribution, clearance, and potential accumulation of CDs in tissues also require deeper investigation. While many studies report efficient renal clearance for small, hydrophilic CDs, others show organ accumulation or retention, especially with surface modifications or metal doping [44]. Comprehensive toxicokinetic studies, including chronic exposure models, are still limited, although they are crucial for establishing safety thresholds necessary for clinical use.

Further, although several studies have demonstrated the ability of CDs to localize in the nucleus and interfere with DNA repair, such as inhibiting BER enzymes [15], the mechanisms of these interactions under physiological and pathological conditions (e.g., hypoxia, inflammation) remain poorly understood. This gap is particularly relevant for their use in cancer therapy, where oxidative microenvironments and impaired DNA repair coexist.

The development of CD-based theranostics, integrating diagnostic and therapeutic functions into a single platform, is conceptually attractive but raises significant formulation and regulatory challenges. For example, the co-delivery of CDs with DNA-damaging agents or chemotherapeutics such as doxorubicin or cisplatin requires precise control of loading efficiency, release kinetics, and synergistic redox activity

Future research should also address their actions in complex biological microenvironments, such as the hypoxic and acidic environment of tumors or the inflammatory environment of degenerative diseases. Understanding how these contexts modulate the oxidative and genotoxic potential of CDs is crucial for their safe and effective use. Encouragingly, recent studies have started to employ machine learning and computational modeling to predict CD behavior and optimize their design for specific biomedical outcomes. These tools may help correlate synthetic parameters with biological functions, accelerating the development of CDs with tailored redox behaviors [86,87].

Finally, standardization across synthetic protocols, physicochemical characterization, and in vitro/in vivo evaluation is urgently needed. Establishing reporting standards analogous to those adopted in pharmacology and toxicology will be fundamental in transitioning CDs from bench to bedside.

## 6. Conclusions

Carbon dots hold great potential as innovative tools for the detection and modulation of nucleic acid oxidative damage. Their unique optical properties, biocompatibility, and ease of functionalization make them ideal candidates for integration into next-generation biosensors and gene-based therapeutic strategies. However, several aspects require further investigation to advance their clinical translation. These include the need for standardized protocols to assess their toxicity, long-term biostability, and the impact of photodegradation on cellular systems.

Moreover, future studies should focus on the rational design of surface chemistry to enhance molecular selectivity, minimize undesired interactions, and exploit CDs’ redox properties in integrated diagnostic and therapeutic (theranostic) approaches.

The convergence of CD technology with aptamer-based sensing and ROS-responsive systems represents a promising direction for developing targeted diagnostic and therapeutic platforms. Future integration with nucleic acid technologies may further enhance their applicability in precision oncology and redox medicine.

Despite these promising applications, challenges remain, including the need to finely control the redox output of CDs to avoid off-target toxicity and the requirement for scalable, reproducible synthesis methods to ensure consistent biological performance. Nonetheless, the inherent versatility and tunability of CDs make them highly promising candidates for redox-based anticancer nanotherapies.

## Figures and Tables

**Table 1:** Toxic doses observed for various functionalized CDs on different types of cells. Copyright (2016), reprinted from [46] with permission from Elsevier. License number 6075790486648.

Entry	Cell Line	Type of CDs	Surface Coverage	Toxic Dose [mg/mL]	Ref.
1	Human kidney cells 293T	Electrooxidation of graphite	Pristine	4	[47]
2	Human liver hepatocellular cells HepG2	Nitric acid oxidation of carbon soot	Pristine	1	[48]
3	Human primary glioblastoma cells U87	Hydrothermal treatment of spider silk	Pristine	2	[49]
4	Human breast cancer cells MCF-7	Nitric acid oxidation of carbon soot	Pristine	0.2	[50]
5		Nitric acid oxidation of carbon soot	PEG1500	0.2	[50]
6		Carbonization of polymeric NPs	PEG1500	0.2	[51]
7		Carbonization of polymeric NPs	PEI	0.015	[51]
8		Carbonization of polymeric NPs	PPE-EI	0.03 *	[51]
9		Carbonization of polymeric NPs	PPEI-EI	0.08 * ^#^	[52]
10		Nitric acid oxidation of carbon soot	PPEI-EI	0.8	[52]
11	Human colorectal adenocarcinoma cells HT-29	Nitric acid oxidation of carbon soot	Pristine	0.2	[50]
12		Carbonization of polymeric NPs	PEG1500	0.2	[50]
13		Carbonization of polymeric NPs	PEG1500	0.2	[51]
14		Laser ablation	PEG1500	0.05 *	[51]
15		Carbonization of polymeric NPs	PPE-EI	0.05	[51]
16		Carbonization of polymeric NPs	PEI	0.015	[51]
17		Nitric acid oxidation of carbon nanopowder	PPEI-EI	0.8 ^#^	[52]

Notes: toxic dose 1⁄4 viability lower than 50%, * higher doses were not tested, ^#^ MTT did not work.

## Data Availability

Not applicable.

## Abbreviations

The following abbreviations are used in this manuscript:

AGT	alkylguanine DNA alkyltransferase
8-OHdG	8-hydroxy-2′-deoxyguanosine
8-oxo-dG	8-oxo-2′-deoxyguanosine
8-oxoG	8-oxoguanine
AFM	Atomic force microscopy
Ag@CDs	silver-CDs nanoconjugates
Au@CDs	gold-CDs nanoconjugates
BER	Base Excision Repair
CAT	catalase
cdA	5′,8-cyclo-2′-deoxyadenosine
cdG	5′,8-cyclo-2′-deoxyguanosine
CDs	Carbon dots
CDs-PEG	PEG-modified CDs
CDs-PEI	polyethyleneimine-coated CDs
CDs-Pri	pristine CDs
CDs–Fe	iron-doped CDs
CDs@ZIF-8	CDs encapsulated within metal–organic frameworks
CNDs	Carbon NanoDots
CPDs	Carbon Polymer Dots
cPu	cyclopurines
CQDs	Carbon Quantum Dots
CS	Cockayne syndrome
DLS	Dynamic light scattering
FRET	fluorescence resonance energy transfer
FTIR	Fourier-transform infrared spectroscopy
GPx	glutathione peroxidase
GQDs	Graphene Quantum Dots
MMR	mismatch repair
NER	Nucleotide excision repair
ROS	Reactive Oxygen Species
SB-HHD-CDs	*Scutellaria barbata* and *Herba Hedyotis diffusae*–based CDs
SOD	superoxide dismutase
TA	Transient absorption
TEM	Transmission electron microscopy
UV	Ultraviolet
XPS	X-ray photoelectron spectroscopy
